# Efficacy of shared decision-making on treatment adherence of patients with bipolar disorder: a cluster randomized trial (ShareD-BD)

**DOI:** 10.1186/s12888-018-1686-y

**Published:** 2018-04-13

**Authors:** L. Samalin, M. Honciuc, L. Boyer, I. de Chazeron, O. Blanc, M. Abbar, P. M. Llorca

**Affiliations:** 1 CHU Clermont-Ferrand, Department of Psychiatry, University of Clermont Auvergne, EA7280 Clermont-Ferrand, France; 2 Aix-Marseille University, Public Health, Chronic Diseases and Quality of Life research Unit, EA 3279 Marseille, France; 3 CH Caremeau, Nîmes, France

**Keywords:** Bipolar disorder, Shared decision-making, Decision aid, Maintenance treatment, Medication adherence, Cluster randomized trial, Study protocol

## Abstract

**Background:**

Shared decision-making (SDM) is a model of interaction between doctors and patients in which both actors contribute to the medical decision-making process. SDM has raised great interest in mental healthcare over the last decade, as it is considered a fundamental part of patient-centered care. However, there is no research evaluating the efficacy of SDM compared to usual care (CAU), as it relates to quality of care and more specifically treatment adherence, in bipolar disorder (BD).

**Methods/Design:**

This is a 12-month multi-centre, cluster-randomized controlled trial comparing the efficacy of SDM to CAU. Adult BD patients (*n* = 300) will be eligible after stabilization for at least 4 weeks following an acute mood episode. The intervention will consist of applying the standardized SDM process as developed by the Ottawa Hospital Research Institute in order to choose the maintenance treatment of BD. A multidisciplinary team developed a decision aid “choose my long-term treatment with my doctor” for BD patients to clarify possible therapeutic options. Primary outcome will assess the patient’s level of adherence (based on hetero-evaluation) of ongoing treatment at 12 months. Secondary outcomes will assess the difference between the 2 groups of patients in terms of adherence to maintenance drug therapy based on other measures (self-assessment scale and plasma levels of mood stabilizers). Additionally, other dimensions will be assessed: decisional conflict, satisfaction with care and involvement in decision making, beliefs about treatment, therapeutic relationship, knowledge about information for medical decision and clinical outcomes (depression, mania, functioning and quality of life).

The primary endpoint will be analysed without adjustment by comparison of adherence scores between the two groups using Student *t*-tests or Mann–Whitney tests according to the variable distribution. A set of secondary analyses will be adjusted for covariates of clinical interest using generalized linear mixed regression models.

**Discussion:**

This will be the first study evaluating the effect of an SDM intervention on patient adherence in BD. This is also an innovative protocol because it proposes the development of an evidence-based tool that should help patients and clinicians to initiate discussions regarding the use of BD treatment.

**Trial registration:**

The study has been registered with ClinicalTrials.gov as NCT03245593.

## Background

Bipolar disorder (BD) is a frequent and severe mood disorder affecting multiple dimensions of the patient’s life: mood, thinking or behaviour [1]. With three million people suffering from BD in Europe [2], the disorder is among the leading causes of disability in the developed world. Further, it is a significant economic burden for patients and the health-care system [3]. BD is a lifelong episodic illness, characterized by periods of recurrence and remission. The variable course of the disorder can often result in functional and cognitive impairment and a reduction in quality of life [4, 5].

Medication treatment represents the first-line therapy for the acute phase and the long-term prophylactic management of BD [6]. However, the ideal preventive strategy should combine pharmacological treatment, adjunctive psychotherapy and lifestyle approaches from the first episode [7]. Moreover, in spite of substantial evidence on effective medications for the treatment of BD, patient outcomes continue to be impacted by lack of treatment adherence [8]. It is estimated that treatment non-adherence occurs at a rate between 12% and 64% among BD individuals [9, 10]. Poor adherence increases the probability of relapse, use of emergency psychiatric services, number of hospital admissions and suicide risk, and compromises clinical and functional outcomes [9, 10].

In 2002, France adopted a bill on patients’ rights and quality of the healthcare system which permitted the official installation of “democracy in healthcare” as “any individual has the right to be informed on his health status” [11]. This provided a legal foundation enabling transition from a paternalistic model to a shared decision-making (SDM) framework. In the following years, French institutions such as the National Health Authority (High Authority of Health) defined SDM model as “a decision support model allowing information sharing between doctors and patients concerning different care options and taking into account patient preferences” [12].

SDM can be regarded as a model of interaction between doctors and patients that aims at changing the asymmetry between the two actors of treatment. This is accomplished by strengthening both the exchange of information and the decisional position of the patient [13]. SDM has raised great interest in mental healthcare over the last decade, as it is considered a fundamental part of patient-centered care.

Positive significant correlations have been established between SDM interventions and improved knowledge, patient participation, and satisfaction with care in schizophrenia and major depressive disorder [13–15]. Even though there is some evidence of SDM interest in these mental disorders, these findings may not readily generalized to BD [15]. As treatment addresses two distinct and sometimes co-occurring sets of symptoms (depression and hypomania/mania), one may wonder if BD patients are not expected to differ from other mental health users in terms of preferences and experience of involvement in treatment decision-making [16].

In the current state of knowledge, only observational studies are available about BD patients’ preferences and decision-making difficulties. Research highlights that BD patients tend to prefer an active collaborative decision-making role and greater levels of involvement than what they are currently experiencing [15]. Several positive outcomes have been associated with collaborative care, in the form of reduced suicide risk and increased patient satisfaction [15]. To our knowledge, there is no research evaluating the use of decision aids in an SDM process to facilitate the involvement of patients in choosing BD treatment in clinical practice. Consequently, there is a great need to assess the efficacy of SDM on quality of care and, more specifically, on treatment adherence and prognostic in BD.

### Aims and hypothesis

We hypothesize that the implementation of a standardized process of SDM during initiation of maintenance treatment will promote better medication adherence in BD patients. Moreover, we argue that BD patients benefitting from SDM will improve their satisfaction with care, psychosocial functioning and quality of life and, further reduce recurrences caused by non-adherence.

The primary objective of the present study is to evaluate the effect of an SDM intervention compared to care as usual (CAU) on maintenance treatment adherence at 12 months following an acute mood episode.

## Methods/Design

### Study design

This is a 12-month multi-centre, cluster randomized controlled trial that will be conducted at 10 public French hospitals.

The Ile de France 1 ethical committee (2017-juil.-14,602 ND) has approved the study procedures described herein. The trial design and protocol are registered with ClinicalTrials.gov as NCT03245593.

**Fig. 1:**
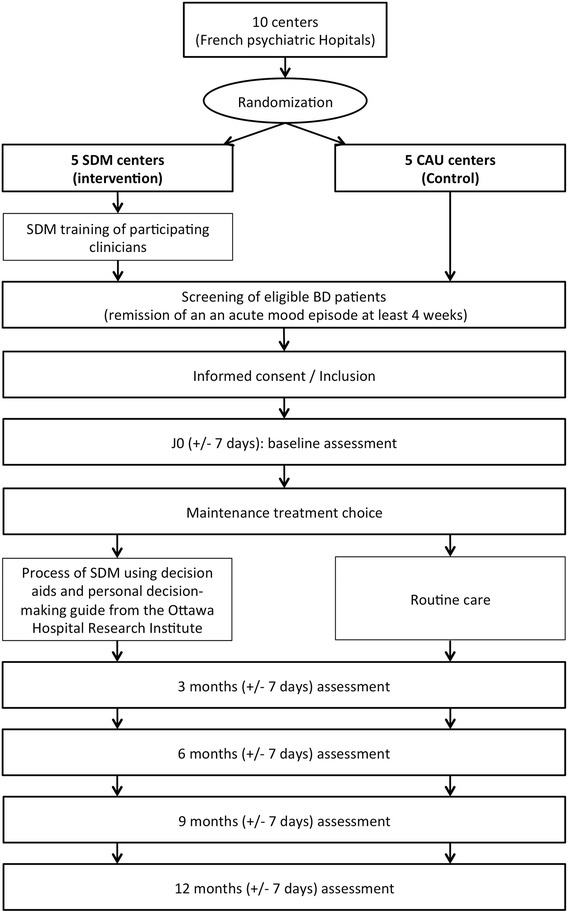
Trial flow diagram

Figure 1 provides an overview of the trial flow.

### Participants

Adult BD patients will be eligible after stabilization of an acute mood episode for at least 4 weeks. Eligible patients will be identified in advance during their hospitalisation or from the appointment schedules of participating practitioners.

Inclusion criteria:

- Age ≥ 18
- DSM-5 criteria for a diagnosis of BD (including Type I and II)
- Remission of an acute mood episode for at least 4 weeks
- Available for 12 months of evaluations
- Able to provide written informed consent
- Comfortable with speaking/reading French
- Member or beneficiary of a French health insurance plan

Non-inclusion criteria

- Participation in another interventional study (currently or in the last 3 months)
- Exclusion period determined by a previous study
- Severe substance use disorders (as defined by DSM-5 criteria)
- Safeguard of justice/guardianship/curatorship
- Difficult to provide informed consent or patient refusing signature of consent

Exclusion criteria

- Withdrawal of consent during study
- Revision of the initial diagnosis of BD

### Allocation procedures

We will randomly assign centers (cluster randomization) to either the intervention arm (SDM centre) or the control arm (CAU centre). Randomization will be performed using SAS software (SAS Institute, Cary, NC, USA) by the methodologist of the study (LB).

Given the study nature, participants, practitioners and investigators cannot be blinded.

For this study, practitioners will assure follow-up and care while investigators will be carrying out regular assessments.

### Intervention

#### Standardised SDM process

The intervention will consist of applying the standardized SDM process as developed by the Ottawa Hospital Research Institute [17] in order to choose the maintenance treatment of BD. Practitioners will deliver support to the decision-making process using the following strategies: 1. clarify decision-making needs; 2. facilitate access to evidence-based information using specific information tools; 3. evaluate comprehension; 4. identify values and prioritize what is important for every participant; 5. strengthen participants’ ability to deliberate, communicate and seek support; 6. monitor and encourage progress in decision-making.

A decision aid (see below) will be presented and discussed with the participants. Then the decision-making process will be continued by using the French version of the personal decision-making guide from the Ottawa Hospital Research Institute [18]. This personal guide provides structured assistance in deliberating on available therapeutic options and communicating with others. It is organized in sequential stages. First, patients are invited to clarify the decision that needs to be made. This involves discussing with the practitioner the decision type and arguments for it, time deadline and patient’s stand-point (e.g. active deliberation, imminent decision or decision already made). Second, patients are encouraged to explore all possible options. Already acquired knowledge is discussed and possible options are assessed according to previously known benefits and risks. Importantly, the personal value of every benefit and risk is assessed for all possible options. Furthermore, implication of those involved with patient (e.g. caregiver) are taken into consideration; their preferences are rated and any perceived pressure by patients reviewed. All these steps are crucial in determinating every person’s needs for decision: knowledge, personal values, entourage support and certainty of his/her choice. Next steps are designed in order to respond to every patient requirement. If adequate knowledge is lacking, diverse approaches are suggested (e.g. information research, question lists, access to evidence-based information). Considering personal value as sometimes difficult to determine, exchange with other patients who already made their decision might be a key solution. Discussing options with trusted persons, sharing the guide with family or friends allows patients to feel encouraged and approved.

The SDM process will begin at inclusion, and the number and frequency of required consultations will be established for each patient by the practitioner. At the end of this SDM process, the ongoing treatment (continuation treatment) will be modified and the maintenance treatment will be initiated. During follow-up the SDM process will be repeated each time adjustments in maintenance treatment are needed.

#### Development of decision aid for BD

The project scientific committee developed, step-by-step, a decision aid entitled “Choose my long-term treatment with my doctor” for BD patients to clarify possible therapeutic options.

The development of this decision aid has been based on a synthesis of evidence regarding the risks and benefits of medications and non-pharmacological therapeutic options in the maintenance treatment of BD. This review was based on the available guidelines, articles or information from the French pharmacovigilance centers and national agencies (French National Agency for Medicines and Health Products Safety, Centre de Réference sur les Agents Tératogènes or CRAT). The CRAT is the first national and international public organization especially involved in the problem of drugs during pregnancy [19].

The construction of the decision aid has been guided by recommendations from the International Patient Decision Aid Standards (IPDAS) [20]. The initial versions of the decision aid have been elaborated from discussions with members of the scientific committee, practitioners and BD patients. Testing of these versions with BD patients has been carried out and the decision aid progressively upgraded.

**Fig. 2:**
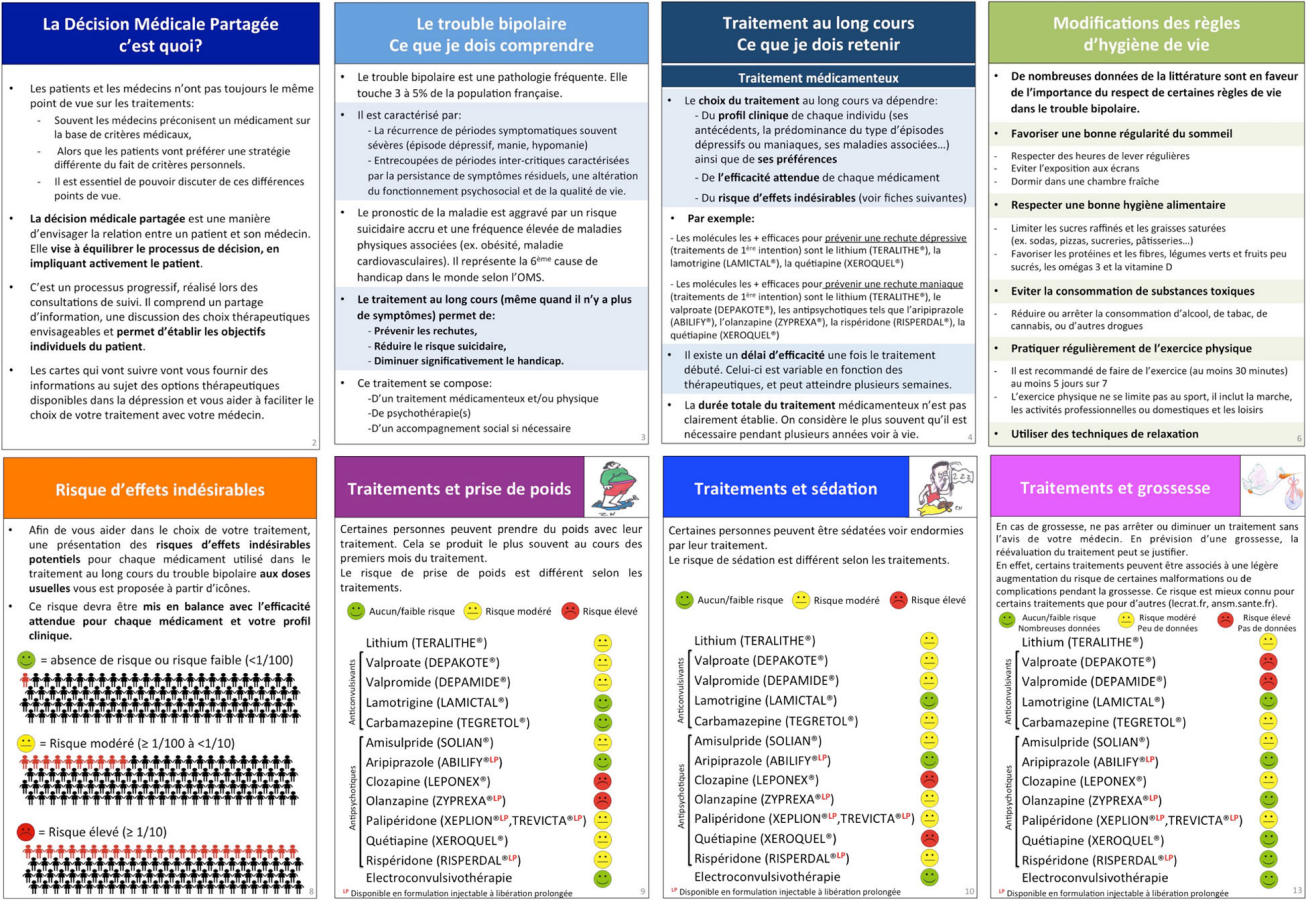
Examples of cards from the decision aid “choose my long-term treatment with my doctor”. From left to right and top to bottom: The Shared-Decision Making, what is it? (Card 2); The bipolar disorder, what do I need to understand? (Card 3); Long-term treatment, what do I need to remember? (Card 4); Lifestyle changes (Card 6); Risk of side-effects (Card 8); Treatments and weight gain (Card 9); Treatments and sedation (Card 10); Treatments and pregnancy (Card 13)

The final version of the decision aid reached consensus among participants involved in this project. It consists of a set of cards offering information about the SDM process, the BD, the main therapeutic options available in term of benefits and risks (i.e. side effects), the main drug interactions, the monitoring of treatment and references of websites or book to give additional information (Fig. 2). Probabilities of risks (none/low < 1%, moderate ≥1% to < 10%, high > 10% using “smiley”) of specific side-effects (weight gain, sedation, hyperprolactinemia and sexual effects, neurologic effects, and other effects) and of complications/teratogenicity during pregnancy for each medication are also detailed.

#### Practitioners/investigators training

All the practitioners of the SDM centres participating in the study will be trained in the SDM approach through participation in a half-day workshop. A maximum of 15 participants per session will be accepted in order to assure their active participation. At the beginning of training, practitioners will receive a complete portfolio including all information received during workshops. As the objectives of the training are comprehension of the study’s design and the acquisition of specific tools and competences needed to implement the standardized process of SDM, various teaching supports will be employed. A three-step organization of training will be employed.

First, practitioners will benefit from a complete overview of the research project including the presentation and discussion of the investigation and the SDM concept followed by presentation of the decision aid for patients and the personal decision support guide (using the tutorial provided by the Ottawa Hospital Research Institute) [17].

Second, participants will acquire practical experience by watching and discussing a video clip demonstrating the process of SDM and basic use of the decision aid with a BD patient. They also will use role-play scenarios of real-life patients encountered in clinical practice.

Finally, at the end of each workshop, an evaluation of command of the information will be carried out, to ensure adequate competences for DMP process implementation.

### Control

In the CAU centers (control arm), practitioners will manage the discussion about the maintenance treatment as usual. They will not have access to the decision aid and will be not trained with the SDM approach.

### Data collection

In this study, offered care and its assessment will be considered as two different processes carried out by distinct professionals: practitioners (psychiatrists delivering care) and evaluators (psychiatrists carrying out assessment). This method will provide sufficient time for practitioners to use the SDM tools: the decision aid and the personal decision-making guide.

Practitioners will include BD patients during a routine consultation. This will occur 4 weeks after the remission of mood episode. They will collect socio-demographical and clinical data: gender, age, family and socio-professional status, level of education, age of onset of bipolar disorder, history of mood episodes (number and duration), number of hospitalizations, ongoing treatment, physical or psychiatric associated comorbidities.

Further, to assess the clinical feasibility of the SDM process, practitioners will note at the end of each consultation the ongoing treatment and consultation duration.

### Outcomes measures

At all time-points identical data will be collected (baseline, 3 months, 6 months, 9 months and 12 months).

#### Primary outcome

The main objective of the project is to evaluate the effect of an SDM intervention compared to CAU on maintenance treatment adherence at 12 months following an acute mood episode. The primary outcome will be assessed by the Clinician Rating Scale (CRS), a standardized and validated hetero-evaluation scale designed to evaluate the patient’s level of adherence to ongoing treatment [21, 22]. The scale ranges from 1 to 7, with higher scores indicating higher adherence (complete refusal, partial refusal, reluctance, partial reluctance, passive acceptance, moderately active acceptance, active participation). The CRS already has been used in clinical trials with BD patients [23, 24] and the French version already has been used in previous studies [25, 26].

#### Secondary outcome

As outlined above, there is an interest in studying the difference between the 2 groups of patients in terms of adherence to maintenance drug therapy based on other measures (self-assessment scale and plasma levels of mood stabilizers). Additionally, other dimensions will be assessed: decisional conflict, satisfaction with care and involvement in decision-making, beliefs about treatment, therapeutic relationship, knowledge about information for medical decision, clinical outcomes (depression, mania, functioning and quality of life) and feasibility of SDM processes in clinical practice.

1. The most reliable measurement of patient’s adherence is a multi-method approach that combines feasible self-reporting and reasonable objective measures [27]. Self-evaluation of medication adherence, collection of plasma levels of ongoing mood stabilizers and record of cancelled consultations also will be collected to improve reliability of the patient’s adherence measure. Patients will complete the Medication Adherence Rating Scale (MARS), a self-assessment scale consisting of 10 items assessing behaviours, attitudes and treatment experience. The psychometric properties of this scale are considered satisfactory in BD patients [28] and its French version has been validated [29].
2. Decisional conflict. Patients will complete the Decision Conflict Scale, a self-assessment questionnaire already validated in French and adequate in measuring patient’s perceptions of uncertainty in the choice of a therapeutic option, modifiable factors contributing to this uncertainty but also to an effective decision-making [30, 31]. It is a 16-items scale, with each item rated from 0 to 4 depending on the degree of agreement or disagreement.
3. Satisfaction with care and involvement in decision-making. As there is no validated French scale available for measuring satisfaction with treatment and the decision-making process for outpatients, a questionnaire has been adapted using the Hospitalized Patient’s Satisfaction Scale and the Clinical Decision-making Involvement and Satisfaction Scale (CDIS) [32, 33]. The latter is a new instrument that considers three items on satisfaction with care, and another three items on satisfaction with the medical decision-making process. All items are rated on a scale of 1 to 5, depending on the degree of agreement or disagreement.
4. Beliefs about treatment. Patients will complete the Beliefs about Medicines Questionnaire (BMQ), a validated 18-items scale meant to assess beliefs about medications in patients with chronic disorder [34]. Its psychometric properties are adequate and the French version has already been validated in populations of patients suffering from BD, schizophrenia or major depressive disorder [35, 36]. The questionnaire includes two different scales: a specific scale assessing patient’s beliefs about the specific treatment of the disease being studied, and a general scale evaluating general beliefs about drugs.
5. Knowledge. Patients will complete the Knowledge questionnaire, a tool developed by the Ottawa Research Institute [37] and adapted for BD patients to assess patient’s levels of knowledge considered essential for making medical decisions. Every item can be rated and total score is calculated by converting true answers into percentages.
6. Another aspect of interest will be the evaluation of clinical outcomes in the two groups. During each visit, various aspects of the BD will be assessed: 1. number, duration and type of mood episodes; 2. number and duration of psychiatric hospitalizations; 3. depressive and manic symptoms severity; 4. quality of life and functioning.
  Depressive symptomatology will be assessed using the French version of the Montgomery-Asberg Depression Rating Scale (MADRS) [38, 39]. This 10-item scale rates various domains such as mood, sleep, appetite, physical and psychic fatigue and ideas of suicide with a score between 0 and 60. Patients also will complete the French version of the Young Mania Rating Scale (YMRS), a validated 11-item scale to assess manic symptoms, with a total score between 0 and 60 [40, 41].
  Psychosocial functioning will be assessed using the Functioning Assessment Short Test, an instrument designed to assess the main functioning problems experienced by psychiatric patients, particularly BD patients [42, 43] This 24-item scale evaluates impairment or disability in six specific areas: autonomy, occupational functioning, cognitive functioning, financial issues, interpersonal relationships and leisure time. The total score varies between 0 and 72, and a score of 11 or higher defines overall functional impairment.
  Patients’ well-being and quality of life will be evaluated using the 26-item version of the World Health Organization (WHO) Quality of Life scale (WHOQOL-BREF). This questionnaire covers 4 dimensions of health-related quality of life: physical health (seven items), psychological health (six items), social relationships (three items), environment (eight items), and two global HRQoL items assess an individual’s overall satisfaction with life and a general sense of personal well-being. The scale has good psychometric properties and has been validated in French. All items are rated on a scale of 1 to 5, and total scores range between 0 (worst) and 100 (best) [44].
7. To evaluate feasibility of the SDM process implementation, consultation duration will be measured. Practitioner satisfaction and decisional conflict will be assessed using the questionnaires previously described (the Decision Conflict Scale and the adapted questionnaire for Satisfaction with care and involvement in decision making).

### Sample size calculation

The sample size was calculated using PASS Power Analysis and Sample Size Software 15.

The number of subjects required was determined from the assumptions based on the primary outcome, the adherence score measured from the CRS (score rated between 1 and 7).

A minimum difference of 1 point between the two groups is considered as clinically relevant (minimal clinically observable change) [21, 22]. A sample size of 5 clusters per group with 23 individuals per cluster achieves 90% power to detect a difference of 1 between the two group means when the standard deviation is 1.4 and the intra-cluster coefficient is 0.05 using a two-sided T-test with a significance level of 0.05 [45, 46]. To prevent 20% of dropout at 12 months, 30 individuals per cluster is necessary and a total of 300 patients should be included.

### Statistical analyses

The data will be analysed using SPSS version 17.0 software and SAS/STAT® version 9.2.

The primary (i.e., adherence CRS score) and secondary (i.e., decisional conflict, satisfaction with care, involvement in decision making, beliefs about treatment, therapeutic relationship, knowledge about information for medical decision and clinical outcomes) endpoints will be analysed on the individual patient level using cluster level summaries, which will account for the correlation between patients in the same ward. The endpoint scores will be compared between control and intervention arms. No interim analysis is planned.

The primary analysis will be performed on the intention-to-treat population corresponding to all included individuals in each participating wards. A secondary analysis will be performed on the per-protocol population corresponding to all included individuals who complete the CRS questionnaire.

Descriptive analyses will provide wards and patient characteristics for each group (control and intervention) at baseline. All analyses will account for clustering to ensure correct type I error rates and confidence intervals [47]. Appropriate statistical methods to account for clustering between patients in the same cluster will be performed using linear mixed-effects models or generalized estimating equations with inclusion of random effects for individual wards and assessment of possible confounding.

The primary endpoint (CRS at 12 months) will be analysed without adjustment by comparison of CRS scores between the two groups using Student t-tests or Mann–Whitney tests according to the variable distribution. A set of secondary analyses will be adjusted for covariates of clinical interest using generalized linear mixed regression models including the group (control/intervention), wards and individuals (as random variables), and other potential confounding variables. These models will be performed using the MIXED SAS procedure (version 9.2). The same procedure will be performed on the secondary endpoints.

Generalized linear mixed regression models will be performed to assess the sustainability of the effect by comparison of monthly means of CRS scores collected during period of the study.

## Discussion

To date, a number of studies have evaluated SDM interventions for several mental disorders (mainly in schizophrenia and major depressive disorder). Most of them have showed a positive effect on satisfaction with care and involvement in decision-making [13–15]. Only one randomized controlled study has evaluated the effectiveness of a collaborative care program (comprised several interventional modules including contracting, psychoeducation, problem-solving treatment or monitoring of outcomes) on symptoms and medication adherence in BD patients experiencing depressive symptoms, in comparison with CAU [48]. The intervention group showed significant reduction of depressive symptom severity at 12 months (*p* = 0.004), with no effect on treatment adherence. However, this collaborative care program included only in part an SDM process; it was not a specific-SDM intervention.

To our knowledge, this will be the first study evaluating the effect of an SDM intervention on patient adherence in BD. We expect that SDM intervention will improve satisfaction with care and involvement in decision making, as previously described, and will improve beliefs about treatment, depressive and manic symptoms severity, relapse rates, functioning and quality of life. These, then, will be addressed as secondary outcomes.

It also will be interesting to know the mean duration of SDM consultation and practitioner satisfaction and decisional conflict, to reduce one of the potential barriers of its implementation in clinical practice. Indeed, systematic review reported that time constraints and lack of applicability due to patient characteristics or clinical situations were the main barriers associated with the implementation of SDM in practice [49]. This last result suggests practitioners might be screening a priori which patients will benefit from SDM and thereby misjudge their preference for active involvement in decision-making.

From our perspective, this is an innovative protocol because it proposes the development and use of a new specific tool in BD management. The decision aid “Choose my long-term treatment with my doctor” for BD patients is an evidence-based tool that should help patients and clinicians initiate discussions regarding the use of BD treatment. This is the first decision aid specifically developed for BD patients. Futures enhancements could be cultural adaptation and development of online supports (e-health or m-health tools).

Potential methodological limitations of this research may be related to the design of the study and the practitioners implementing the intervention. To reduce bias due to the open design of the study and minimize the burden of the trial procedures, care and its assessment will be considered as two different processes carried out by distinct professionals: practitioners (psychiatrists delivering care) and evaluators (psychiatrists carrying out all the assessments). Cluster randomization will be carried out at centre levels in order to avoid effects of contamination due to uncontrolled diffusion of knowledge acquired by professionals benefitting from the SDM decision aid training.

If there is an increased interest of SDM in the decision-making process of patients with mental disorders, it has not yet been widely adopted by mental health professionals. The development of a decision aid for BD patients should facilitate implementation of SDM in clinical practice. We could hope that this proposal research – via its primary and secondary outcomes – will reduce the medical burden associated with BD.

## Abbreviations

BD: Bipolar disorder
BMQ: Beliefs about medicines questionnaire
CAU: Care as usual
CDIS: Clinical decision-making involvement and satisfaction scale
CRS: Clinician rating scale
HGLM: Hierarchical generalized linear model
IPDAS: International patient decision aid standards
MADRS: Montgomery-Asberg depression rating scale
MARS: Medication adherence rating scale
SDM: Shared decision making
WHOQOL-BREF: World Health Organization Quality of Life scale (short-version)
YMRS: Young mania rating scale
